# Angiogenic factor-driven improvement of refractory thin endometrium with autologous platelet-rich plasma intrauterine infusion in frozen embryo transfer cycles

**DOI:** 10.3389/fendo.2024.1431453

**Published:** 2024-09-03

**Authors:** So Yeon Shin, Nanum Chung, Ji Eun Shin, Jee Hyun Kim, Chan Park, Hwang Kwon, Dong Hee Choi, Jung Ryeol Lee, Ji Hyang Kim

**Affiliations:** ^1^ Fertility Center, CHA Bundang Women’s Medical Center, CHA University Bundang Medical Center, Seongnam, Republic of Korea; ^2^ Department of Obstetrics and Gynecology, Seoul National University Bundang Hospital, Seongnam, Republic of Korea; ^3^ Department of Translational Medicine, Seoul, Republic of Korea; ^4^ Department of Obstetrics and Gynecology, Seoul National University College of Medicine, Seoul, Republic of Korea

**Keywords:** platelet-rich plasma, refractory thin endometrium, endometrial receptivity, implantation, embryo transfer, angiogenesis

## Abstract

**Objective:**

A beneficial effect on endometrial thickness (EMT) and improvement of pregnancy outcome after intrauterine infusion of platelet-rich plasma (PRP) has been suggested. This study assessed the effect of intrauterine PRP infusion on live birth rate and obstetrical outcomes and analyzed cytokines that can potentially improve pregnancy outcomes through PRP.

**Method:**

This study was a prospective cohort study conducted in a university hospital fertility center. The study included ninety-one patients who had a history of two or more failed *in vitro* fertilization (IVF) attempts and refractory thin endometrium that remained unresponsive after at least two conventional treatments for thin endometrium. Patients were treated with an intrauterine infusion of autologous PRP between days 7 and 14 of their hormone replacement therapy-frozen embryo transfer (HRT-FET) cycle. PRP was administered at 3-day intervals until their EMT reached 7mm. After a maximum of three PRP administrations, embryo transfer (ET) was performed. The primary outcome was the live birth rate. Secondary outcomes included the implantation rate and increase in EMT compared to the previous cycle. We compared the cytokines related to angiogenesis in a patient’s whole blood (WB) and PRP by utilizing a commercial screening kit.

**Results:**

The live birth rate in the PRP treatment cycle was 20.9% (19 of 91 patients), significantly superior to the previous cycle without PRP infusion (p < 0.001). The implantation rate was also significantly higher during the PRP treatment cycle (16.4%) compared to the previous cycle (3.1%) (p < 0.001). The mean EMT post-PRP treatment was 6.1 mm, showing a significant increase of 0.8 mm (p < 0.001). Nonetheless, an increase in EMT was also observed in the non-pregnancy group. No adverse effects were reported by patients treated with autologous PRP. Cytokine array analysis confirmed marked increases in well-known pro-angiogenic factors such as Ang-1, EGF, LAP (TGF-b1), MMP-8, PDGF-AA, and PDGF-AB/PDGF-BB.

**Conclusion:**

Intrauterine PRP infusion offers a safe and effective treatment for patients with refractory thin endometrium and implantation failures. The angiogenic cytokines present in PRP are the primary drivers of this improvement.

## 1 Introduction

For successful embryo implantation, good-quality embryos and a receptive endometrium are required. Despite significant advances in *in-vitro* fertilization (IVF), embryo culture, and freezing-thawing techniques, thin or damaged endometrium remains a challenge for successful implantation. Thin endometrium is defined as <7mm in thickness on the day of human chorionic gonadotropin (hCG) trigger in a fresh IVF cycle, or on the day to start progesterone in frozen-thaw embryo transfer (FET) cycle (1). An endometrium thickness (EMT) of less than 7mm is assumed to be suboptimal for embryo implantation (2). Several treatments, including exogenous estrogen, vitamin E, vaginal sildenafil, and pentoxifylline, were clinically used but did not result in a definitive response from patients with a refractory thin endometrium (3).

Platelet-rich plasma (PRP) has been extensively used as a cutting-edge technique for regeneration in different fields of medicine. PRP has been applied to incurable diseases such as musculoskeletal injuries, diabetic foot ulcers (4), erectile dysfunction (5), cartilage degeneration (6), alopecia (7), and skin rejuvenation (8), which are diseases that do not have many treatment options.

A beneficial effect on EMT after intrauterine infusion of PRP was first suggested by Chang and colleagues in 2015 (9–11). Recently, emerging data have shown that increased EMT and improvement in pregnancy rate were observed after intrauterine infusion of autologous PRP in patients with thin endometrium (9–11).

Platelets are known to be the blood component that plays a crucial role in hemostasis. PRP contains hundreds of bioactive molecules such as growth factors, cytokines, and cell adhesion molecules within platelet α granules (12). PRP has immuno-modulatory (13), anti-inflammatory (14), and angiogenic effects (15, 16) and also promotes tissue repair.

Our preliminary pilot study involving 22 patients highlighted potential improvements in implantation, pregnancy, and live birth rates following PRP use. However, its limited sample size precluded definitive conclusions (17). Other previous studies have shown the benefits of PRP treatment on refractory thin endometrium (10, 11, 18), but few have investigated the underlying mechanism of this treatment. Therefore, in this study, we aimed to assess the effect of intrauterine PRP infusion on pregnancy and implantation in a larger cohort. We also analyzed the pregnancy rate and implantation rate results according to the etiology of a thin endometrium and present the possible mechanism of PRP treatment with cytokine analysis of PRP. Furthermore, we investigated maternal and neonatal adverse effects over an extended observation period that exceeded 1 year.

## 2 Materials and methods

### 2.1 Study population and inclusion criteria

We conducted an interventional prospective cohort study. Patients were recruited from December 2015 to February 2021 in a single fertility center of a university hospital. The concept and procedures of this study were approved by the Bundang CHA Hospital Institutional Review Board (IRB number: 2015-10-181), and all patients provided written informed consent. Women who had a history of two or more failed IVF cycles and refractory thin endometrium were enrolled in this study. We included 100 patients with the following criteria: (a) age of 20–45 years at the time of enrollment, (b) endometrial thickness (EMT) of <7 mm on the hCG administration day in fresh embryo transfer (ET) cycles or at the end of estrogen priming day in frozen ET cycles in two or more previous cycles, (c) two or more failed IVF cycles, (d) more than two cycles of previous therapy for increasing the EMT, such as hysteroscopic adhesiolysis following hormone replacement therapy, high dose estradiol valerate, transvaginal sildenafil administration, or pentoxifylline combination with vitamin E, (f) frozen embryo available for ET, and (g) informed consent form signed.

Of 100 patients, we excluded 9 patients due to the following exclusion criteria: (a) hematologic disorders, hemoglobin (Hb) level of <10.0 g/dL or (b) platelet count of <100,000/μL, (c) chromosomal abnormality in the patient or spouse, (d) body mass index (BMI) of ≥30 kg/m2, and (f) uncontrolled endocrine or other medical conditions, such as hyperprolactinemia or thyroid diseases.

### 2.2 Autologous PRP preparation

On each day of PRP administration, 18 mL of venous blood was collected from the patients using 30 mL syringes coated with 2 cc of acid citrate dextrose solution A (NOTHROM Soln.; DAE HWA Pharm. Co., Ltd., Seoul, Korea), an anticoagulant solution. The blood samples were then transferred to a sterile PRP centrifuge kit (PROSYS PRP; Prodizen, Korea) and centrifuged at 3000rpm for 3 min. Approximately 0.3~0.4 mL of PRP was generated by collecting the buffy coat and plasma just above the buffy coat. Based on the data provided by the manufacturer, the platelet concentration of the PRP ranged from 717 × 103 to 1565 × 103/μL, and the WBC concentration varied from 24,000 to 37,000/μL.

### 2.3 Autologous PRP administration and ET

Intrauterine autologous PRP administration was performed during the estrogen-primed hormone replacement treatment-FET (HRT-FET) cycle. The patients started taking 6 mg of estradiol valerate (Progynova; Bayer Schering Pharma, France) daily from the 2nd day of their menstrual cycle (MCD #2) to prepare the endometrium. During the endometrial preparation process, additional agents such as sildenafil, pentoxifylline, vitamin E, granulocyte colony stimulating factor (G-CSF), or aspirin were not used. The first autologous PRP injection was performed on MCD #10 and repeated at 3-day intervals until the EMT reached 7 mm. PRP was administered into the uterine cavity using a 5Fr feeding tube (F.D.T(D), HMS Inc, Korea) connected to a 1cc syringe containing activated PRP catheter within 1 h of completion of PRP preparation. Activated PRP was prepared by the mixture of 1/10 of the amount of buffy coat with calcium gluconate (calcium gluconate hydrate 0.1042g/ml) just before the PRP insemination. The syringe containing the PRP was filled with air and connected to the feeding tube. The air filled approximately 0.15 to 0.2cc of the remaining space in the syringe and was used to push the remaining PRP. The completion of PRP infusion was confirmed with the air bubble under ultrasonography. The maximum number of autologous PRP infusions was limited to three. Ultrasonography was performed to measure the EMT on MCD#2 and every autologous PRP administration day until ET. ET was conducted 3 days after the final autologous PRP administration. After a maximum of three times intrauterine PRP administrations, ET was performed with either day 3 or day 5 embryos, even if the EMT did not reach 7mm. Luteal phase support was performed using either daily 90 mg of vaginal progesterone (Crinone gel 8%; Merck, Germany) or 50 mg of progesterone (Taiyu Progesterone Inj. Jaytech Biogen, South Korea) administered via intramuscular injection daily from 3 to 5 days before the ET day according to the cultured stage of embryos. Estradiol valerate was continuously administered during luteal phase support. To control the potential confounding factors, the intrauterine PRP infusion and ET procedures were conducted by the same physician.

Serum β-hCG levels were measured in peripheral blood 9 or 11 days after ET. Those with positive β-hCG results underwent an ultrasound scan 2 weeks later to confirm clinical pregnancy. Clinical pregnancy was defined as the presence of an intrauterine gestational sac. Luteal phase support was continued until 9 weeks of gestation. The obstetric progress of the pregnant patients was tracked through timely chart reviews and questionnaires.

### 2.4 Cytokine analysis of PRP

After obtaining PRP from a random individual using the method described previously, the PRP was prepared for cytokine screening analysis, along with a whole blood sample from the same patient. Employing the Proteome Profiler Array Kit (ARY007, Human, Angiogenesis, R&D Systems), all procedures were followed according to the manufacturer’s protocol. Additionally, we used the Quick Spots Tool (Western Vision Software, Version 22.0.1b, analyzed by WOONGBEE MeDiTech, Inc.) to gather raw data on spot density. This dataset was subsequently subjected to quantitative analysis, enabling a comparative assessment of the cytokines expressed within both the whole blood and PRP samples.

### 2.5 Statistical analysis

Statistical analysis was performed using SPSS (IBM corporation, version 26). Mean ± standard deviation was used to describe normally distributed data. Paired t-test was used for comparison between pre-PRP and post-PRP treatment. One-way ANOVA was conducted to compare pre- or post-PRP treatment EMT according to ET outcome. Kolmogorov-Smirnov normalization test and Wilcoxon-ranked test were performed to analyze the patients’ etiology and treatment outcome. Statistical significance was set at P<0.05.

## 3 Results

A total of 100 women were recruited and nine were excluded. Of the latter, two withdrew from the study before PRP treatment, three were unable to undergo embryo transfer because no embryos were obtained for transfer, two had Hb<10.0g/dL, one was over 45 years old, and another had a BMI>30.

The patients’ baseline characteristics are summarized in **Table 1**. Total pregnancy rate including biochemical pregnancy with a threshold of β-hCG >5 mIU/mL, clinical pregnancy rate, implantation rate, and live birth rates were significantly increased after PRP treatment. EMT was increased after intrauterine PRP infusion (**Table 2**). Pre-PRP treatment EMT referred to the thickness on the day of hCG trigger in the most recent IVF cycle or the EMT on the day the patient started progesterone in their adjacent previous FET cycle. Post-PRP treatment EMT was measured on the day of embryo transfer after a maximum of three intrauterine PRP infusions with three days apart. The clinical pregnancy rate after PRP treatment was 31.9% (29/91), and the live birth rate was 20.9% (19/91). Live birth rate (P <0.001) and clinical pregnancy rates (P <0.001) were significantly increased after PRP treatment. Among the 29 clinically conceived patients, 10 patients had a miscarriage. One of the patients with pregnancy loss had a heterotopic pregnancy, and the intrauterine fetus was aborted at 8 weeks of gestation after laparoscopic removal of the ectopic tubal mass.

**Table 1:** Baseline characteristics of included patients.

Variable	Data (N = 91)
Age (years)	38.6 ± 3.9
BMI (kg/m^2^)	22.1 ± 2.9
No. of previous curettages	1.4 ± 1.4
Failed IVF cycles	3.16 ± 3.7
Duration of infertility (years)	4.21 ± 2.7
Parity (n)	
Nulliparous Parous	76 15
Baseline platelet count(µL)	258k ± 55.6
No. of transferred embryos	2.3 ± 0.8
No. of good quality embryos transferred	1.1 ± 0.8
No. of patients for PGT-A	12
Endometrial thickness	
Pre-PRP treatment	4.97 ± 1.21

BMI, body mass index; PGT-A, Preimplantation genetic test for aneuploidy; PRP, Platelet-rich plasma.

Good quality embryo: defined as grade I or II cleavage-stage embryo with six or more cells, Morula grade I or II by Veek’s qualification scale, and blastocyst 3BB or higher in Gardner scoring system.

**Table 2:** Pregnancy outcome and endometrial thickness following infusion of platelet-rich plasma.

Variable	Pre-PRP treatment cycle	Post-PRP treatment cycle	*P* value Difference (95% CI)
Embryos			
Transferred embryos (n)	2.24 ± 0.85	2.30 ± 0.80	0.712
Good quality embryos (n)	0.79 ± 0.98	1.05 ± 0.82	0.078
Pregnancy			
Total pregnancy rate (%)	14.3% (13/91)	42.9% (39/91)	< 0.001
Implantation rate (%)	3.1% (4/128)	16.8% (37/220)	< 0.001
Clinical pregnancy rate (%)	3.3% (3/91)	31.9% (29/91)	<0.001
Live birth rate (%)	0% (0/91)	20.9% (19/91)	< 0.001
Endometrium			
Endometrial thickness (mm)	5.0 ± 1.2*	6.1 ± 1.3*	< 0.001 (0.89 – 1.42)

*Data all represent the mean ± standard deviation. Total pregnancy rate included both clinical pregnancy and chemical pregnancy. Significant differences are accepted for P < 0.05. Clinical pregnancy was defined as intrauterine gestational sac identification with fetal heart activity on ultrasound examination 4 to 5 weeks after embryo transfer.

The etiology of thin endometrium was subcategorized into three groups. The first group was patients with a history of mechanical endometrial trauma such as curettage, myomectomy, or septotomy. The second group was patients with thin endometrium with no known causation, referred to as idiopathic, and the third group was categorized as other causes, including patients with a past history of uterine artery embolization, diagnosed with pelvic tuberculosis, with a past history of pelvic inflammation, or with uterine anomaly. We compared the increase in EMT and clinical pregnancy rate according to the etiology (**Table 3**). More than half of the patients (n=65, 71.4%) had a history of iatrogenic trauma such as curettage, myomectomy, or septotomy, and the idiopathic group had the second most patients (n=20, 22.0%). There was no statistical difference in pregnancy rate according to the etiology of thin endometrium.

**Table 3:** Etiology of thin endometrium in this study.

Etiology	N	EMT before PRP infusion	EMT after PRP infusion	*P (95% CI)*	Δ EMT	Patients with good quality embryos (N)	Clinical pregnancy rate (%)
Idiopathic	20	4.7 ± 1.1*	5.8 ± 1.4*	0.003 (0.42 - 1.70)	1.1 ± 1.4*	65.0% (13/20)	30.0% (6/20)
Traumatic	65	5.1 ± 1.2*	6.2 ± 1.3*	<0.001 (0.79 - 1.41)	1.1 ± 1.3*	73.8% (48/65)	33.8% (22/65)
Other cause UAE Pelvic Tb PID Uterine anomaly	6 1 1 3 1	3.93 ± 0.73* 3.00 3.10 4.50 ± 0.21* 4.00	5.98 ± 0.79* 6.00 6.20 6.10 ± 0.94* 5.40	0.027^+^ N/A N/A N/A N/A	2.05 ± 1.08* N/A N/A 1.60 ± 1.14* N/A	–	16.7% (1/6) 0/1 0/1 1/3 0/1

N, number; EMT, endometrial thickness; Δ EMT = EMT after PRP infusion – EMT before PRP infusion; *Data all represent mean ± SD. Kolmogorov-Smirnov normalization test, P is measured by paired t-test. No difference in the rate of good quality embryo and clinical pregnancy rate between idiopathic and traumatic etiology by chi-square test (p= 0.57 and p=0.75). Kruskal-Wallis test for comparing EMT before PRP, after PRP, and Δ EMT. Significant differences are accepted for p < 0.05.

In total, 14 term deliveries with 16 babies including two twin pregnancy patients, and five preterm deliveries were reported. The obstetrical outcomes can be seen in **Table 4**. The mean gestational age at delivery was 37.3 ± 3.2 weeks, and the mean neonatal weight at the time of delivery was 2.8 ± 0.8 kg. Most of the live births, 88.9%, were delivered by cesarean section. Placenta accrete spectrum (PAS) disorder was shown in 4 out of 19 cases. Among these, two patients experienced postpartum hemorrhage defined as blood loss greater than 1000ml during cesarean section or more than 500ml within 24h after the vaginal delivery (19). One patient was persistently monitored with an ultrasound scan for the retained placenta for more than 6 months postpartum. One patient delivered the fetus at 23 weeks because of the rupture of the amniotic membrane. The fetus was delivered and expired after NICU care (**Table 4**).

**Table 4:** The obstetric outcome of patients with live birth after PRP treatment.

Patient No.	Age	GA	Neonate Wt(kg)	NICU Adm.	Delivery mode	Pl. accreta	Postpartum hemorrhage	Complication
1	35	38 + 6	3.7	–	c/sec(CPD)	+	–	–
2	39	39 + 2	3.3	–	VD	–		–
3	40	37 + 2	3.3	–	c/sec(Ut. scar)	–	–	–
4	31	40 + 0	3.00	–	c/sec(CPD)	+	+	Hypomenorrhea
5	34	36 + 6	3.2	–	c/sec(unknown)	–	–	–
6	42	39 + 0	3.4	–	VD	–	–	–
7	37	39 + 6	2.8	–	c/sec(CPD)	–	–	–
8	40	38 + 0	3.1	–	c/sec(Ut. scar)	–	–	AUB
9	38	38 + 0	2.6/2.6	–	c/sec(twin)	–	–	AUB
10	37	38 + 0	3.1	–	c/sec(Br)	–	–	–
11	42	27 + 2	0.8	+	c/sec(preterm)	–	–	–
12	38	36 + 3	2.3/2.3	+	c/sec(PIH)	–	–	–
13	37	28 + 4	1.2	+	c/sec(PIH)	–	–	–
14	31	38 + 4	2.8	–	c/sec(Br)	+	–	–
15	40	38 + 2	3.2	–	c/sec(CPD)	+	+	Retained placenta, hypomenorrhea
16	37	40 + 1	3.7	–	c/sec (fetal distress)	–	–	–
17	41	23	n/a	+	n/a	–	–	Baby Expired
18	35	40 + 0	3.4	–	c/sec(CPD)	–	–	–
19	35	term	n/a/n/a	–	c/sec(twin)	–	–	–
Count [N(%)] Or mean ± SD	37.3 ± 3.2	Term:14 preterm: 5	2.8 ± 0.8	4/19 (21.1%)	16/18 (88.9%)	4/19 (21.1%)	2/19 (10.5%)	

GA, gestational age at the delivery; Wt, weight; NICU Adm., Neonate Intensive Care Unit admission; Pl. accrete, placenta accreta; n/a, not available; c/sec, Cesarean Section; VD, Vaginal delivery; CPD, Cephalopelvic disproportion; Ut. Scar, previous uterine scar; Br, Breech; PIH, Pregnancy induced hypertension; AUB, Abnormal uterine bleeding.

**Figure 1:**
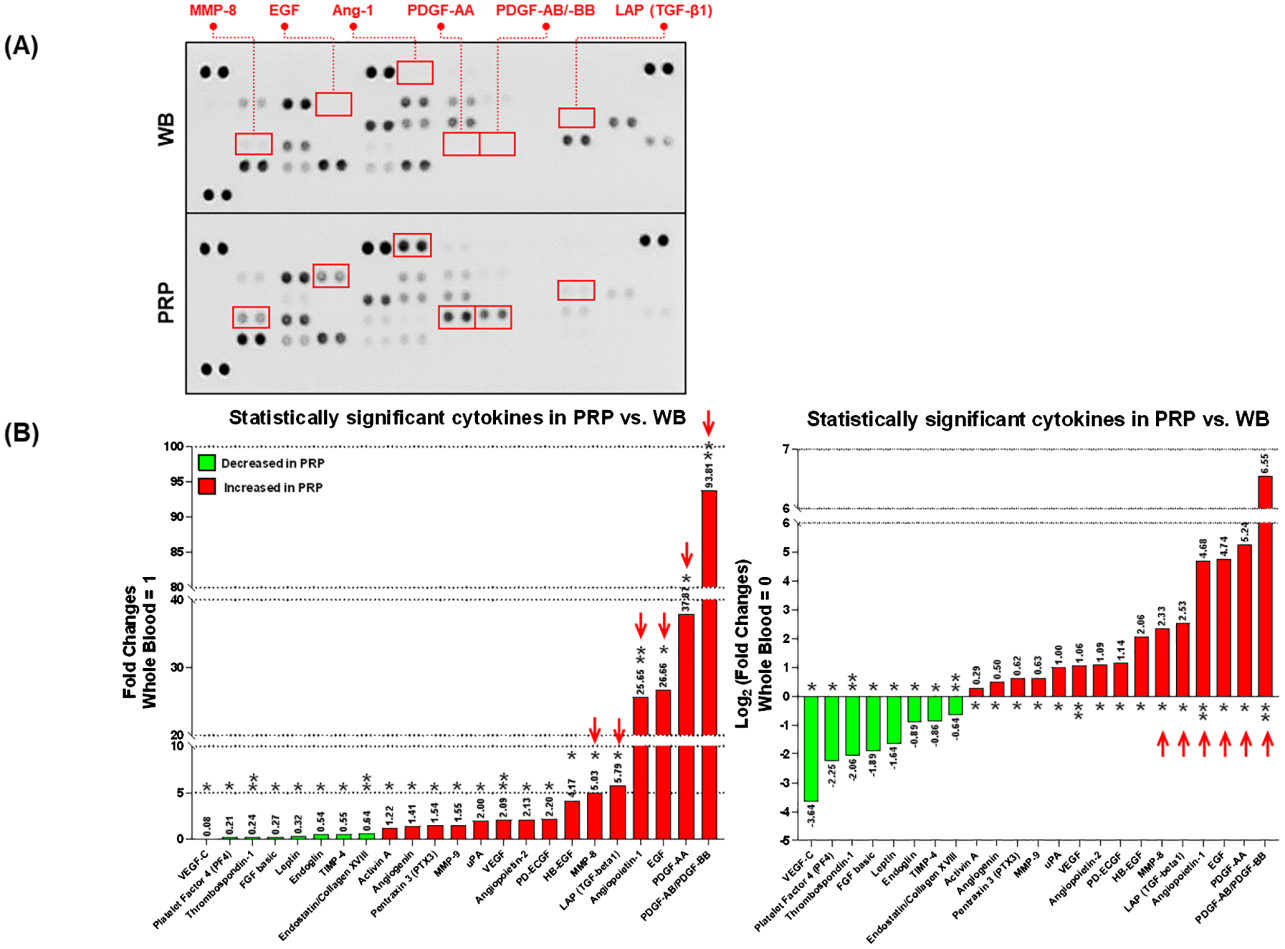
The comparison of angiogenesis-related cytokines between the platelet-rich plasma (PRP) and whole blood (WB). **(A)** The blotted membranes of angiogenesis-related cytokines in WB and PRP. The paired blots highlighted by red squares indicate the cytokines (connected labels), which are relatively increased in the PRP compared to the WB (>5-fold). **(B)** Only the statistically significant cytokines (total of 55) in PRP are exhibited in this figure, by relatively comparing their FC (left) and log2 transformation of FC (right) values to those in WB. Red arrows in each graph indicate the cytokines detected over 5-fold more in the PRP compared to the WB. Each annotation above or below the bars shows the plotted values, and asterisks indicate the statistical significance (*p<0.05, and **p<0.01). PRP, Platelet-rich plasma; WB, Whole blood; FCs, Fold changes; VEGF, Vascular endothelial growth factor; FGF, Fibroblast growth factor; TIMP, Tissue inhibitor of metalloproteinase; MMP, Matrix metalloproteinase; LAP (TGF-β1), N-terminal latency-associated peptide (LAP) of the transforming growth factor β1 (TGF-β1) dimer; uPA, Urinary plasminogen activator; PD-ECGF, Platelet-derived endothelial cell growth factor; Ang-1, Angiopoietin-1; HB-EGF, Heparin-binding EGF-like growth factor; EGF, epidermal cell growth factor; PDGF, Platelet-derived growth factor.

In total, 23 cytokines in PRP exhibited significant differences compared to the whole blood (Number of decreased cytokines=8, and increased cytokines=15). Notably, matrix-mellatoproteinase-8 (MMP-8; 5.03 folds), N-terminal latency-associated peptide (LAP) of the transforming growth factor β1 (TGF-β1) dimer (LAP(TGF-β1); 5.79 folds), angiopoietin-1 (Ang-1; 25.65 folds), endothelial growth factor (EGF; 26.68 folds), platelet-derived growth factor (PDGF)-AA (37.87 folds), and PDGF-AB/BB (93.81 folds) were significantly increased in PRP, more than 5-fold compared to the whole blood (**Figures 1A, B**).

## 4 Discussion

This study is the result of data extension of the previous pilot study of 20 women who underwent IVF-frozen cycles with intrauterine administration of PRP to improve pregnancy outcomes (17). In this relatively larger study population, we ascertained improved live birth rate, implantation rate, and an increase in endometrial thickness. To the best of our knowledge, this is the first study to report pregnancy rates and endometrial thickness according to the etiology of thin endometrium and obstetric outcomes and postpartum complications more than 1 year of observation in endometrial PRP treatment.

It is known that thin endometrium is associated with low clinical pregnancy rate and low birth rate, especially if the EMT is less than 7mm (20). In addition, several studies revealed that an increased pregnancy rate was observed when EMT reached 10mm (21). Thin endometrium has been related to reduced growth of glandular epithelium (22) containing secretions of uterine histiotroph, referring a complex array of carbohydrates, proteins, and lipids from the endometrial gland (23). It is also characterized as an aberrant ratio of T cell subsets, immunological factors, and cytotoxicity of uterine natural killer cells (24). Defective vascularization, and in fact, subendometrial blood flow have been associated with EMT (25). The altered blood flow dynamic leads the embryos closer to the spiral arteries, and increased oxygen concentrations and reactive oxygen species (ROS) of the basal layer of the endometrium (26) can affect implantation rate (27).

Although there were no statistically significant differences, we observed a slight increase in the number of good-quality embryos during the treatment cycle. However, the patients enrolled in this study had refractory thin endometrium and had experienced cycle cancellations and implantation failures more than twice in previous cycles, despite treatment with pentoxifylline, sildenafil, aspirin, vitamin E, or G-CSF. We believe that endometrial factors, rather than embryo quality, were the major contributors to pregnancy failures in these cases. Additionally, when we compared the number of good quality embryos before and after PRP treatment within the groups that achieved live birth and the group that did not, we found no statistical difference. In the live birth group, the number of good-quality embryos transferred was 1.3 ± 1.16 in the pre-PRP treatment cycle and 1.3 ± 0.67 in the post-PRP treatment cycle (P=1.000). Similarly, in the non-live birth group, the number of good quality embryos transferred was 1.0 ± 0.99 in the pre-PRP treatment cycle and 0.98 ± 0.84 in the post-PRP treatment cycle, P=0.925). In future research, controlling for embryos using preimplantation genetic testing could address these limitations.

In this relatively larger patient unit, unlike the previous pilot study, EMT was significantly increased after PRP treatment albeit the mean EMT was still below 7mm after treatment. Nevertheless, we found a significantly increased live birth rate compared to the previous cycle. From this result, it was concluded that, in the case of PRP treatment, there would be no need to cancel the embryo transfer even if the endometrial thickness did not reach 7mm. We supposed that PRP infusion not only increased endometrial thickness but also had an effect on functional improvement to build a favorable environment for implantation. PRP is an autologous biomaterial with no known adverse effects and its biology of dense granules is more complex than the pharmaceutical drugs currently in use. The clinical outcome of PRP is the result of the application of various cell constituents in the specimen interplaying with the recipient’s local microenvironment (28). Several potential mechanisms were proposed to explain the improvement of pregnancy and implantation outcomes after PRP treatment.

It has been well documented that PRP initiates tissue repair via the release on the supraphysiologic level of biologically active molecules and adhesion proteins. In the cytokine assay conducted in this study, the proangiogenic factors Ang-1, EGF, MMP-8, LAP(TGF-β1), PDGF-AA, and PDGF-AB/BB were observed to be more than five times higher in PRP compared to whole blood. Ang-1 plays a crucial role in angiogenesis and regeneration, and it is essential for maintaining vascular integrity (29). EGF has a role in the proliferation, differentiation, growth, and migration of epithelial cells and keratinocytes which induce epithelization in granulation wounds and reduce cutaneous scarring in mice (30). MMPs regulate tissue remodeling and angiogenesis through the breakdown of the extracellular matrix (30). Previous studies have reported that PRP increases the expression of MMP-1, MMP-3, MMP-7, and MMP-26 (15). In this study, a significant increase in MMP-8 was observed. MMP-8 is known as the major MMP of neutrophils and primarily degrades collagen types I, II, and III (31). This suggests that collagen deposition might decrease. There was also a research finding from a rat uterine scar model that indicated that the upregulation of MMP-8 promotes endometrium regeneration through collagen degradation (32). LAP(TGF-β1) is the pro-secreting form which is noncovalently associated with mature TGF-β before secretion. TGF-β1 is an important regulator of smooth muscle cell and endothelial cell growth (33). A knock-out mice model for the TGF-B family revealed their critical importance in angiogenesis (34, 35). PDGF is a glycoprotein with two disulphide-bonded polypeptides, referred to as A and B chains. There are three isoforms, PDGF-AA, -BB, and –AB (36). PDGF seems to be the first growth factor present in a wound and initiates connective tissue healing through the promotion of collagen and protein synthesis (37). Considering the elevated levels of key proangiogenic factors in PRP, it is evident that PRP may significantly enhance endometrial receptivity by promoting angiogenesis. This enhancement is likely through promoting vascular integrity, stimulating epithelial proliferation and migration, facilitating extracellular matrix remodeling, and initiating connective tissue healing, collectively contributing to endometrial regeneration and improved implantation.

It was confirmed that the pregnancy rate, implantation rate, and live birth rate increased regardless of the presence and the degree of increase in the EMT (**Supplementary Table 1**). Although there was an increase in EMT, it did not increase by more than 7mm. Therefore, it is believed that the increase in implantation and pregnancy rate was not simply due to the increase in thickness but likely due to an improvement in the endometrial function. As previously mentioned, growth factors and regulating molecules jump-start healing in chronic injuries and accelerate the repair process in acute injuries via endocrine, paracrine, autocrine, and intracrine mechanisms of the healing process.

We analyzed the causes of thin endometrium to determine which patient groups benefitted significantly from PRP treatment. We validated the treatment outcome based on the etiology of thin endometrium to specify the treatment indication for PRP. However, when evaluating the pregnancy rates by etiology, no statistically significant differences were observed between the groups. Therefore, it is believed that PRP can increase the endometrial thickness and improve the pregnancy rate regardless of the etiology. Additionally, the group categorized as others appeared to show a relatively greater increase in endometrial thickness, but the relative pregnancy rate was observed to be lower. Nevertheless, this observation was not statistically significant due to the limited sample size.

Placenta accreta spectrum (PAS) refers to a clinical condition where the placenta fails to detach spontaneously and requires forceful manual removal. This condition can lead to significant hemorrhaging post-delivery (38). Intrauterine adhesion is recognized as a risk factor for abnormal placentation, which can result in PAS (39, 40). Four out of 19 participants with live births (21.1%) reported adherent placenta. However, none underwent hysterectomy nor received histological confirmation to differentiate whether the condition was placenta accreta, increta, or percreta. The incidence of PAS in this study was higher than the normal population, which is estimated to be 3.7 per 1000 births in the nationwide inpatient sample in the United States (41). However, the result is not very far from the incidence of placenta accreta after hysteroscopic adhesiolysis of intrauterine adhesion or Asherman’s syndrome, which is reported to be between 19.3% and 23% (42, 43), while considering more than half of patients had intrauterine adhesion confirmed by hysteroscopy.

After PRP treatment, 43 patients were monitored for a period ranging from 6 to 28 months, excluding those who became pregnant or were lost to follow-up. During this follow-up period, patients underwent symptom monitoring such as abnormal vaginal bleeding and ultrasound examinations. Additionally, 19 of the patients received hysteroscopic surgery and EM biopsy. Throughout the observation period, no adverse events from PRP treatment, including endometrial hyperplasia or malignant transformation were indicated by ultrasound or hysteroscopic biopsies. Additionally, ultrasound examinations did not reveal abnormal endometrial thickening in any of the patients.

This study recruited a relatively larger number of patients compared to the previous pilot study. Thus, we could derive statistical significance from increases in live births, clinical pregnancy, implantation rate, and EMT. The strength of our study is that it is the first study to report pregnancy rate and endometrial thickness according to the etiology of thin endometrium and obstetric outcomes and postpartum complications in more than 1 year of observation after treatment. The cytokine assay revealed elevated levels of angiogenic factors in PRP, indicating a potential mechanism for these observed improvements. However, the study was limited by its design as a single-arm, prospective cohort study without a control group. Nevertheless, there is significance in this study that this patient cohort underwent multiple IVF trials and remained unable to achieve pregnancy over an average infertility period of more than 4 years. Additionally, we confirmed that angiogenic factors were increased in PRP compared to whole blood. However, we were unable to collect endometrial tissue or endometrial fluid at the time of embryo transfer. We believe that future studies are necessary to demonstrate the proangiogenic effect of PRP in the human endometrium.

## 5 Conclusion

Autologous PRP treatment can enhance the clinical pregnancy rate, implantation rate, and endometrial thickness without adverse effects in patients with refractory thin endometrium. From our results, PRP is also associated with an improved live birth rate and does not significantly increase complications such as placenta accreta. Moreover, cytokine assays have indicated a substantial elevation in proangiogenic factors within PRP, which may elucidate the mechanism behind its therapeutic benefits. To establish PRP as a standard treatment for thin endometrium, further research must focus on understanding the precise mechanisms in detail and conducting robust, well-designed randomized controlled trials with larger cohorts. Achieving a consensus on the standardization of PRP preparation, the ideal number of infusions, and precise dosages, is essential. Additionally, refining the indications for PRP use will optimize its impact on pregnancy outcomes.

## Data Availability

The original contributions presented in the study are included in the article/**Supplementary Material**. Further inquiries can be directed to the corresponding authors.
