# Supplementary material for: Angiogenic factor-driven improvement of refractory thin endometrium with autologous platelet-rich plasma intrauterine infusion in frozen embryo transfer cycles
Source: Front Endocrinol (Lausanne). 2024 Sep 3;15:1431453. doi: 10.3389/fendo.2024.1431453 (PMC11405219; doi:10.3389/fendo.2024.1431453)
Supplement: Supplementary file 1 [file Table1.docx]

**Supplementary Table 1** outcome of embryo transfer and change in endometrial thickness

| Variable | No. of patients(N=91) | EMT before PRP treatment | EMT after  PRP treatment | Δ EMT | *P* value  Difference (95% CI) |
| --- | --- | --- | --- | --- | --- |
| Failed pregnancy | 52(57.1%) | 5.1±1.3 | 6.1±1.4 | 1.0±1.3 | < 0.001(0.64-1.38)^+^ |
| Biochemcial pregnancy | 10(11.0%) | 4.9±1.1 | 5.9±1.6 | 1.1±1.2 | 0.024(0.18-1.92)^+^ |
| Clinical pregnancy | 29(31.9%) | 4.8±1.0 | 6.2±1.1 | 1.5±1.2 | <0.001(1.00-1.90)^+^ |
| live birth | 19(20.9%) | 4.7±1.0 | 6.2±1.1 | 1.5±1.1 | <0.001(0.98-2.02)^+^ |
| miscarriage | 10(11.0%) | 5.0±1.1 | 6.3±1.3 | 1.4±1.4 | 0.013(0.37-2.34)^+^ |

Biochemical pregnancy: b-hCG>5mIU/mL. Data all represent mean ±SD. Kolmogorov-Smirnov normalization test.

*P*=0.507 for one-way ANOVA for comparing pre-treatment EMT of patients in failed pregnancy, biochemical pregnancy and clinical pregnancy, *P*=0.804 for one-way ANOVA for comparing post-treatment EMT of patients in failed pregnancy, biochemical pregnancy and clinical pregnancy, *P*=0.315 for one-way ANOVA for comparing Δ EM thickness. Among clinical pregnancy, Mann-Whitney test for before/after/ Δ EM thickness comparing ongoing pregnancy and miscarriage patients (p=0.228, 0.839, 1.000). ^+^P is measured by paired t-test.
